# Radiation dosimetry of 18F-FDG PET/CT: incorporating exam-specific parameters in dose estimates

**DOI:** 10.1186/s12880-016-0143-y

**Published:** 2016-06-18

**Authors:** Brian Quinn, Zak Dauer, Neeta Pandit-Taskar, Heiko Schoder, Lawrence T. Dauer

**Affiliations:** Department of Medical Physics, Box 84, Memorial Sloan Kettering Cancer Center, 1275 York Avenue, New York, NY 10065 USA; Department of Radiology, Memorial Sloan Kettering Cancer Center, New York, USA

**Keywords:** PET/CT, CT, Radiation exposure, Effective dose, 18F-FDG

## Abstract

**Background:**

Whole body fluorine-18 fluorodeoxyglucose positron emission tomography/computed tomography (PET/CT) is the standard of care in oncologic diagnosis and staging, and patient radiation dose must be well understood to balance exam benefits with the risk from radiation exposure. Although reference PET/CT patient doses are available, the potential for widely varying total dose prompts evaluation of clinic-specific patient dose. The aims of this study were to use exam-specific information to characterize the radiation dosimetry of PET/CT exams that used two different CT techniques for adult oncology patients and evaluate the practicality of employing an exam-specific approach to dose estimation.

**Methods:**

Whole body PET/CT scans from two sets of consecutive adult patients were retrospectively reviewed. One set received a PET scan with a standard registration CT and the other a PET scan with a diagnostic quality CT. PET dose was calculated by modifying the standard reference phantoms in OLINDA/EXM 1.1 with patient-specific organ mass. CT dose was calculated using patient-specific data in ImPACT. International Commission on Radiological Protection publication 103 tissue weighting coefficients were used for effective dose.

**Results:**

One hundred eighty three adult scans were evaluated (95 men, 88 women). The mean patient-specific effective dose from a mean injected 18F-FDG activity of 450 ± 32 MBq was 9.0 ± 1.6 mSv. For all standard PET/CT patients, mean effective mAs was 39 ± 11 mAs, mean CT effective dose was 5.0 ± 1.0 mSv and mean total effective dose was 14 ± 1.3 mSv. For all diagnostic PET/CT patients, mean effective mAs was 120 ± 51 mAs, mean CT effective dose was 15.4 ± 5.0 mSv and mean total effective dose was 24.4 ± 4.3 mSv. The five organs receiving the highest organ equivalent doses in all exams were bladder, heart, brain, liver and lungs.

**Conclusions:**

Patient-specific parameters optimize the patient dosimetry utilized in the medical justification of whole body PET/CT referrals and optimization of PET and CT acquisition parameters. Incorporating patient-specific data into dose estimates is a worthwhile effort for characterizing patient dose, and the specific dosimetric information assists in the justification of risk and optimization of PET/CT.

## Background

Positron emission tomography/computed tomography (PET/CT) has become an indispensable imaging modality for the diagnosis, staging and monitoring of therapy response of a broad range of malignancies [1, 2]. PET/CT is a valuable tool in oncology due to the combined metabolic and morphological information provided. The PET emission scan provides physiological information using a set of detectors that are independent of CT transmission detection, and although two independent PET and CT images may be coregistered to form a single image, the two scans are most accurately coupled when both are acquired during the same exam using a combined PET and CT scanner. For this reason, and because a built-in CT provides other conveniences such as attenuation correction, most modern PET scanners are dual PET/CT units. Regardless of acquisition circumstances, the patient dose from PET and the patient dose from CT in PET/CT exams are first estimated separately in different ways, and then combined to give a total whole body radiation dose. Referral for PET/CT studies must be justified in each case as a first general principle of radiological protection [3]. Optimization, or ensuring that the diagnostic information is as high as reasonably achievable while maintaining radiation doses as low as reasonably achievable, is the second general principle in radiologic protection according to the International Committee on Radiological Protection (ICRP) [4]. Additionally, increased awareness of the risk of exposure to ionizing radiation has resulted in efforts to minimize radiation dose incurred during x-ray and nuclear medical imaging tests [5]. Implementation of any dose saving strategies depends critically on accurate dose measurement/dosimetry to maximize the benefit/risk ratio from imaging tests [6].

A reliable method of estimating patient dose, which includes appropriate units of dose measurement and appropriate calculation methods, is important for any exam involving ionizing radiation. As Stabin stated, the biokinetic model used to calculate the dose is one of the major uncertainties in the evaluation of radiation doses for radiopharmaceuticals [7]. If careful patient specific dosimetry is performed, with attention paid to accurate measurement of individual organ volumes, many of the biokinetic model uncertainties can be minimized, and the total uncertainty in the individual dose estimate can be reduced to perhaps ±10 %-20 %[7]. Effective dose is a parameter that allows a meaningful comparison of the radiation dose from the radiopharmaceutical and the x-ray portions of a PET/CT scan. Effective dose is not directly measured, rather it is calculated based on equivalent doses to organs and the radiosensitivities of the organs. Effective dose is commonly used when evaluating relative biologic risk (*4*), and the Monte Carlo–based organ dose coefficients should not be used to calculate effective dose for individual patients [6, 8]. The International Organization for Medical Physics, ICRP, Health Physics Society and American Association of Physicists in Medicine (AAPM) all have issued policy statements that note the dangers of extrapolating biological risk estimates for radiation doses less than 100 mSv [6, 9–11]. The establishment and use of risk coefficients to estimate public health determinants from individual or population exposures must be considered in the context of uncertainties in the estimates [12]. These uncertainties include dosimetric uncertainties, epidemiological and methodological uncertainties, uncertainties from low statistical power and precision in epidemiology studies of radiation risk, uncertainties in modeling risk data, generalization of risk estimates across different populations and dose rates, as well as reliance on epidemiological studies on observational rather than experimental data [13]. Uncertainties in such risk estimates have been suggested as being up to a factor of 3 lower or higher than the value itself [14]. These rather large uncertainties cause predictions of radiation induced cancers or detriment to be susceptible to biases and confounding influences that are unidentifiable. With due recognition of the limitations imposed by the uncertainties inherent in correlating dose to risk, effective dose is a useful approximation for relating patient dose from internal and external sources, evaluating population characteristics and evaluating optimization efforts. Organ doses from PET are estimated based on the injected activity, while organ doses from CT are estimated based on scanner-specific monte carlo simulations or the dose parameter reported in the exam dose report. Both approaches can be made to more accurately reflect the actual patient dose by including scanner- and patient-specific factors.

Without specific information, risk evaluations may be based on reference or literature values that must be carefully chosen if used for risk evaluation. The International Atomic Energy Agency’s “Radiation Protection of Patients” (RPOP) is a reputable online reference resource that provides information for health healthcare professionals, member states and the general public to achieve safer use of radiation [15]. In the absence of specifics, reference values can overgeneralize and can result in conservative overestimates of dose in an attempt to account for many variables. Such generalization is inherent in non-specific reference values for PET/CT exams because of the number of PET and CT variables contributing to overall dose. CT technique differs greatly if it is just for attenuation correction or if it is for diagnostic-quality images. Attenuation correction can be performed with CT currents as low as 10 mA but most institutions use CT currents of 40-80 mA [16]. Further, an oncology center, for example, may have an intermediate CT technique that provides images with better image quality than a technique only for attenuation correction but lower image quality than a diagnostic CT technique. PET dose varies primarily with injected activity, but like CT dose varies with patient size. The dependency of total dose on many factors warrants a critical review of general reference values and suggests the importance of specific data.

Several approaches to PET/CT dose estimation utilize exam-specific parameters. Dose Length Product (DLP) is a standardized CT dose parameter that is established for each CT scanner using a phantom, equal to CTDIvol multiplied by the length of the scan. DLP can be related to effective dose by multiplying DLP by a patient-specific factor, or scanner-specific monte carlo simulation data can be employed to estimate CT dose [17, 18]. ImPACT method calculates organ dose from CT and uses simplified stylized anatomic models that are anatomically crude but widely used for practical applications with the standard mathematic representations of the reference man and other representative phantoms in radiation protection, nuclear medicine, and medical imaging [19–21]. Radiopharmaceutical organ dose is estimated utilizing conversion coefficients and injected activity, such as those in ICRP 106. Effective dose is typically calculated from both PET and CT organ dose utilizing tissue-weighting factors, such as those in ICRP 103.

In these ways, effective dose from both PET and CT both are calculated and combined to represent a whole body burden. This combined whole body burden is useful in evaluating both the relative and absolute benefit of the combined scan. Although PET/CT is widely performed, published patient dose data is as varied as the parameters determining the dose and often focuses on a narrow patient population [22–31]. The potential for variation in PET scan practices and CT techniques is especially apparent when one considers the wide range of scanner makes & models, injected activity and patient populations in different countries. Many clinic-specific elements that influence radiation dose can be incorporated into the dose estimate to make the dose more specific to the patient.

The aim of the present study was to characterize the radiation dosimetry of two types of routine whole body PET/CT protocols at our institution using patient-specific data and commonly available dose estimation techniques. We compare the results of using patient-specific data with reference and published values and ascertain the utility of such data in the evaluation of risk/benefit for justification and protocol optimization in routine clinical utilization.

## Methods

### Study population

Institutional review board approval with waiver of patient informed consent was obtained to perform a retrospective study of clinically indicated whole body PET/CT scans performed on consecutive adult oncology patients at Memorial Sloan Kettering Cancer Center from January 2010 through December 2011. Patient consent was not required.

### Patient population data

Consecutive adult patients who had undergone PET/CT with either standard registration or full dose diagnostic quality CT techniques for a variety of oncological indications, were identified. Patient demographics are shown in Table 1.

**Table 1:** Patient demographics

	No. patients	Age	Body mass index
		Yrs	
Male	95	53.7 ± 18.1 (18.8–90.2)	26.2 ± 4.8 (15.1–46.1)
Female	88	52.1 ± 20.3 (18.4–96.7)	26.6 ± 7.8 (15.6–71.5)
Male and female	183	52.3 ± 19.3 (18.4–96.7)	26.4 ± 6.4 (15.1–71.5)

All data are mean ± standard deviation (range)

### Exam data

For each PET exam performed, the amount of 18F-FDG administered was obtained from the medical records. For each CT study, image data was reviewed to determine the body region that was being examined, and this was repeated for each CT series. The following parameters for each series were extracted from the DICOM headers, dose reports and scan scout images for later organ equivalent dose and effective dose calculation: (a) kVp, (b) mA, (c) beam collimation, (d) rotation time, (e) pitch, (f) volume CT dose index (CTDIvol) and (g) dose length product (DLP).

### 18F-FDG PET/CT exam

All whole-body PET/CT patients fast for at least 6 h, blood glucose concentration is determined before the injection of the radiopharmaceutical. A nominal injection activity of 12 mCi (444 MBq) is prescribed in our clinic, and +/- 10 % of the prescribed activity is actually injected intravenously into the patient. The actual injected activity is recorded for each patient. Following an approximately 60 min uptake time, PET/CT exams are performed on General Electric (GE) scanners. Data for this study were recorded from patients scanned on Discovery 690 DSTE PET/CT, which utilizes a GE Lightspeed 16 CT scanner (GE Healthcare, Milwaukee, Wis). Tube current modulation is utilized for all CT acquisitions except the “scout” scan, which uses a fixed technique of 120 kVp, 10 mA, pitch 3 and rotation time 0.5 s. The first part of the exam sequence is acquisition of a scout CT image used to select the desired anatomy to be scanned and establish bed positions for PET acquisition. The technologist selects from base of the skull to mid thighs for body PET/CT scans. Next a CT scan is acquired using either the standard or the diagnostic CT technique, depending on the required patient specific protocols completed by the radiologist. The technique for all standard PET/CT exams in this study is 120 kVp, auto-mA, rotation time 0.8 sec, slice thickness 3.75 mm, collimator width 10 or 40 mm, pitch 1.75. The technique for all PET/CT exams with a diagnostic CT is 120 kVp, auto-mA, rotation time 0.6 s, slice thickness 5.00 mm, collimator width 20 or 40 mm, pitch 1.375. CT techniques are summarized in Table 2.

**Table 2:** CT technique summary

CT technique description	Tube potential	Tube current	Revolution time	Collimator width	Pitch	Effective mAs^a^	CTDIvol	DLP	Effective Dose	Effective dose per unit mAs
	(kVp)	(mA)	(s)	(mm)			(mGy)	(mGy cm)	(mSv)	(mSv/mAs)
Standard male	120	78 ± 8.2	0.8	10 or 40	1.75	38.6 ± 10.0	5.1 ± 0.6	464 ± 86	5.3 ± 1.0	0.142 ± 0.026
Standard female	120	72 ± 11	0.8	10 or 40	1.75	39.4 ± 12.4	4.8 ± 0.7	407 ± 73	4.6 ± 0.8	0.124 ± 0.028
Diagnostic male	120	236 ± 86	0.6	20 or 40	1.375	116 ± 40.5	12.1 ± 2.8	1185 ± 249	17.4 ± 3.7	0.164 ± 0.063
Diagnostic female	120	223 ± 109	0.6	20 or 40	1.375	122 ± 59.3	10.2 ± 3.9	912 ± 368	13.4 ± 5.5	0.121 ± 0.039

Data are the mean ± standard deviation

^a^Effective mAs is calculated as [(mA*tube rotation)/pitch]

CT is followed immediately by PET acquisition with a 3-min emission acquisition time per bed position, a 15.3-cm axial field of view per bed position with a 23 % bed overlap.

### Internal radiation-absorbed dose assessment

The OLINDA\EXM code (version 1.1, Vanderbilt University, Nashville, TN, USA) was used to determine the organ equivalent dose and effective dose from each PET series with patient-specific parameters [32]. The biokinetic model parameters as defined in Bolch et al (2009) and specifically identified in ICRP Publication 106 for 18F-FDG were used as input factors for OLINDA [33, 34]. The ICRP biokinetic model for 18F-FDG was derived from data in Hayes et al and Deloar et al [35, 36]. The OLINDA code also allows for the modification of standard reference phantoms to more closely represent patient-specific factors, such as patient weight and corresponding organ mass. We used the standard anthropomorphic models as well as models modified to represent patients weight and height (i.e., organ sizes of the phantom models used by OLINDA were modified to reflect the patient-specific mass of the organ as described by Marine et al and Clark et al [37–39]. Modified adult male, adult female models in OLINDA’s phantom library were utilized to generate patient-specific organ equivalent dose, and then tissue weighting factors from ICRP Publication 103 were used to generate patient-specific effective dose conversion factor (mSv/MBq) [6]. These factors were multiplied by injected activity (MBq) for each PET study to obtain an estimation of effective dose.

### External radiation-absorbed dose assessment

Effective dose from CT examination was estimated using the CT-specific method using the ImPACT spreadsheet, employing ICRP 103 weighting factors [17].

### Statistical analysis

Descriptive and summary statistics were performed with a spreadsheet application (Excel 2007, Microsoft, Redmond, WA). Statistical analysis included average, median, standard deviation, minimum, maximum and range.

## Results

### Study population

Of the total 183 PET/CT scans evaluated, 95 (52 %) were performed on male patients and 88 (48 %) were performed on female patients. Forty-six male patients and 39 female patients received the standard CT technique. Forty-nine male patients and 49 female patients received the diagnostic CT technique. Subjects ranged in age from 18 to 96 years (mean ± standard deviation (SD) 52.6 ± 18.2 years). The subjects’ weight ranged (mean ± SD) from 42.0 to 182 kg (74.9 ± 17.7 kg), height ranged (mean ± SD) from 120 to 198 cm (168 ± 10.3 cm), and BMI ranged (mean ± SD) from 15.1 to 71.5 (26.4 ± 6.4).

### 18F-FDG

The mean ± SD (range) 18F-FDG injected activity for all patients was 454 ± 33.3 MBq (152 to 488 MBq).

### CT techniques

The mean ± SD (range) effective mAs of the standard CT for all patients was 39.0 ± 11.2 mAs (27.4 to 69.4 mAs), 38.6 ± 10.0 mAs (27.4 to 69.4 mAs) for male patients and 39.4 ± 12.4 mAs (27.4 to 69.4 mAs) for female patients.

The mean ± SD (range) effective mAs of the diagnostic CT for all patients was 119 ± 50.6 mAs (27.4 to 231 mAs), 116 ± 40.5 mAs (50.0 to 231 mAs) for male patients and 121.6 ± 59.3 mAs (27.1 to 231 mAs) for female patients.

CT techniques are summarized in Table 2.

### Radiation doses – 18F-FDG

The five organs with the highest organ equivalent doses from 18F-FDG in all patients, in order of highest to lowest dose, were bladder, heart, brain, liver and lungs. 18F-FDG organ equivalent doses are summarized in Tables 3 and 4.

**Table 3:** Organ equivalent dose and effective dose from 18F-FDG PET/CT with standard CT to adult male and female patients

Organ	Dose from 18F-FDG, mGy^a^		Dose from CT, mGy^b^		Total dose, mGy	
	Male (n = 46)	Female (n = 39)	Male (n = 46)	Female (n = 39)	Male (n = 46)	Female (n = 39)
Adrenals	5.1 ± 0.9	6.0 ± 1.0	5.3 ± 1.0	4.6 ± 0.8	10.4 ± 0.9	10.6 ± 0.9
Brain	15.9 ± 3.2	17.3 ± 3.2	6.0 ± 1.0	5.3 ± 0.9	21.9 ± 2.8	22.6 ± 2.9
Breasts	3.6 ± 0.7	4.3 ± 0.8	4.9 ± 0.9	4.2 ± 0.7	8.4 ± 0.8	8.5 ± 0.7
Gallbladder wall	5.4 ± 1.0	6.2 ± 1.8	5.6 ± 1.0	4.9 ± 0.9	11.1 ± 0.9	11.1 ± 1.0
Colon	4.9 ± 0.9	5.9 ± 1.0	5.2 ± 1.0	4.6 ± 0.8	10.2 ± 0.9	10.5 ± 0.9
Small intestine	5.1 ± 0.9	5.7 ± 1.0	5.2 ± 1.0	4.6 ± 0.8	10.3 ± 0.9	10.2 ± 0.9
Stomach wall	4.7 ± 0.9	5.6 ± 1.0	5.7 ± 1.0	4.9 ± 0.9	10.3 ± 0.9	10.5 ± 0.9
Heart wall	29.1 ± 6.2	35.4 ± 6.9	5.9 ± 1.1	5.2 ± 0.9	35.0 ± 5.6	40.6 ± 6.5
Kidneys	4.5 ± 0.8	5.3 ± 0.9	5.8 ± 1.1	5.1 ± 0.9	10.3 ± 0.9	10.3 ± 0.9
Liver	8.9 ± 1.7	11.0 ± 2.0	5.6 ± 1.0	4.8 ± 0.8	14.4 ± 1.4	15.8 ± 1.7
Lungs	8.3 ± 1.7	10.0 ± 1.8	6.3 ± 1.2	5.4 ± 1.0	14.6 ± 1.4	15.4 ± 1.5
Muscle	4.2 ± 0.8	5.0 ± 0.9	3.8 ± 0.7	3.3 ± 0.6	8.0 ± 0.7	8.3 ± 0.7
Ovaries/Testes^c^	4.6 ± 0.8	7.0 ± 1.2	1.1 ± 0.2	4.3 ± 0.8	5.6 ± 0.8	11.2 ± 1.0
Pancreas	5.2 ± 1.0	6.2 ± 1.0	5.2 ± 1.0	4.5 ± 0.8	10.4 ± 0.9	10.7 ± 0.9
Red marrow	4.2 ± 0.9	4.8 ± 1.0	4.4 ± 0.8	3.8 ± 0.7	8.5 ± 0.8	8.6 ± 0.9
Osteogenic cells	6.2 ± 1.8	67.4 ± 2.0	8.0 ± 1.6	6.9 ± 1.2	14.2 ± 1.5	14.3 ± 1.7
Skin	3.2 ± 0.6	3.7 ± 0.7	3.4 ± 0.6	3.0 ± 0.5	6.6 ± 0.6	6.7 ± 0.6
Spleen	4.4 ± 0.8	5.2 ± 0.9	5.4 ± 1.0	4.7 ± 0.8	9.8 ± 0.9	9.9 ± 0.9
Thymus	4.8 ± 0.9	5.7 ± 1.0	6.4 ± 1.2	5.6 ± 1.0	11.2 ± 1.0	11.3 ± 1.0
Thyroid	4.2 ± 0.8	4.4 ± 0.8	8.3 ± 1.4	7.3 ± 1.3	12.4 ± 1.2	11.7 ± 1.1
Urinary bladder wall	59.1 ± 5.2	79.5 ± 6.7	5.5 ± 1.0	4.8 ± 0.8	64.6 ± 4.8	84.3 ± 6.4
Uterus	–	8.8 ± 1.4	–	4.6 ± 0.8	–	13.4 ± 1.2
Effective dose^d^	8.1 ± 1.2	10.1 ± 1.4	5.3 ± 1.0	4.6 ± 0.8	13.4 ± 1.1	14.7 ± 1.2

^a^Patient specific average dose calculated by OLINDA

^b^Patient specific average dose calculated by ImPACT

^c^Ovaries for female patients/Testes for male patients

^d^Effective dose in mSv estimated by ICRP Publication 103

**Table 4:** Organ equivalent dose and effective dose from 18F-FDG PET/CT with diagnostic CT to adult male and female patients

Organ	Dose from 18F-FDG^a^		Dose from CT^b^		Total dose	
	Male (n = 49)	Female (n = 49)	Male (n = 49)	Female (n = 49)	Male (n = 49)	Female (n = 49)
Adrenals	5.0 ± 0.6	5.9 ± 1.1	17.6 ± 3.7	13.5 ± 5.5	22.6 ± 3.4	19.4 ± 5.0
Brain	15.9 ± 2.1	16.6 ± 3.6	21.0 ± 4.4	16.1 ± 6.5	36.9 ± 3.6	32.8 ± 5.5
Breasts	3.6 ± 0.5	4.2 ± 0.9	15.7 ± 3.3	12.0 ± 4.8	19.3 ± 3.0	16.2 ± 4.4
Gallbladder wall	5.4 ± 0.7	6.0 ± 1.2	18.7 ± 3.9	14.4 ± 5.8	24.1 ± 3.1	20.4 ± 5.2
Colon	4.9 ± 0.6	5.8 ± 1.1	17.5 ± 3.7	13.5 ± 5.5	22.4 ± 3.4	19.3 ± 5.0
Small intestine	5.1 ± 0.6	5.6 ± 1.1	17.5 ± 3.7	13.5 ± 5.5	22.6 ± 3.4	19.1 ± 5.0
Stomach wall	4.7 ± 0.6	5.4 ± 1.1	18.6 ± 3.9	14.4 ± 5.8	23.3 ± 3.6	19.8 ± 5.3
Heart wall	29.0 ± 4.1	34.4 ± 7.7	20.0 ± 4.2	15.3 ± 6.2	48.9 ± 3.8	49.7 ± 6.4
Kidneys	4.4 ± 0.5	5.2 ± 1.0	19.1 ± 4.0	14.7 ± 6.0	23.5 ± 3.8	19.9 ± 5.5
Liver	8.9 ± 1.2	10.7 ± 2.2	17.9 ± 3.8	13.8 ± 5.6	26.8 ± 3.3	24.5 ± 4.7
Lungs	8.3 ± 1.1	9.8 ± 2.1	20.0 ± 4.2	15.3 ± 6.2	28.3 ± 3.7	25.1 ± 5.2
Muscle	4.2 ± 0.5	4.8 ± 1.0	12.5 ± 2.7	9.6 ± 3.9	16.7 ± 2.4	14.4 ± 3.4
Ovaries/Testes^c^	4.6 ± 0.6	6.8 ± 1.3	3.5 ± 0.7	12.6 ± 5.1	8.1 ± 0.6	19.4 ± 4.5
Pancreas	5.2 ± 0.6	6.1 ± 1.2	17.2 ± 3.6	13.2 ± 5.3	22.4 ± 3.3	19.3 ± 4.8
Red marrow	4.1 ± 0.6	4.7 ± 1.1	14.6 ± 3.0	11.2 ± 4.5	18.7 ± 2.7	15.9 ± 3.9
Osteogenic cells	6.1 ± 1.2	7.1 ± 2.1	26.4 ± 5.5	20.3 ± 8.2	32.6 ± 4.8	27.5 ± 7.0
Skin	3.2 ± 0.4	3.6 ± 0.7	11.1 ± 2.3	8.6 ± 3.4	14.2 ± 2.1	12.2 ± 3.1
Spleen	5.2 ± 1.08	5.1 ± 1.0	17.4 ± 3.7	13.4 ± 5.4	21.7 ± 3.4	18.5 ± 4.9
Thymus	4.4 ± 0.5	5.5 ± 1.1	21.8 ± 4.6	16.9 ± 6.8	26.6 ± 4.3	22.5 ± 6.2
Thyroid	4.2 ± 0.5	4.3 ± 0.9	28.0 ± 5.8	21.6 ± 8.7	32.2 ± 5.6	25.9 ± 8.2
Urinary bladder wall	59.1 ± 3.7	78.9 ± 9.2	18.5 ± 3.9	14.2 ± 5.7	77.7 ± 4.8	93.1 ± 10.2
Uterus	-	8.6 ± 1.6	-	13.8 ± 5.8	-	22.4 ± 4.9
Effective dose^d^	8.1 ± 0.8	9.9 ± 1.6	17.4 ± 3.7	13.4 ± 5.5	25.5 ± 3.4	23.4 ± 4.9

^a^Patient specific average dose calculated by OLINDA

^b^Patient specific average dose calculated by ImPACT

^c^Ovaries for female patients/Testes for male patients

^d^Effective dose estimated by ICRP publication 103

Mean ± SD (range) patient-specific effective dose from 18F-FDG calculated by OLINDA for all patients was 9.0 ± 1.6 mSv (3.4 to 13.6 mSv), 9.1 ± 1.7 mSv (5.4 to 12.8 mSv) for male and 10.0 ± 1.5 mSv (3.4 to 13.6 mSv) for female. Mean ± SD (range) effective dose per unit injected activity for all patients was 0.0199 ± 0.0032 mSv/MBq (0.0132 to 0.0291 mSv/MBq). 18F-FDG patient-specific effective doses are summarized in Table 5.

**Table 5:** 18F-FDG Dose summary

CT technique description	Injection activity (MBq)		Effective dose (mSv)		Effective dose per unit activity (mSv/MBq)	
	Male	Female	Male	Female	Male	Female
Standard CT	455 ± 30	450 ± 26	8.1 ± 1.2	10.1 ± 1.4	0.018 ± 0.002	0.022 ± 0.002
Diagnostic CT	455 ± 25	454 ± 51	8.1 ± 0.8	9.9 ± 1.6	0.018 ± 0.002	0.022 ± 0.003

Data are the mean ± standard deviation. n, male standard CT=46. n, female standard CT = 39. n, male diagnostic CT = 49. n, female diagnostic CT = 49

### Radiation doses - CT

The mean ± SD (range) effective dose for all patients from the CT portion of scans employing the standard technique was 5.0 ± 1.0 mSv (2.9 to 7.2 mSv), while it was 15.4 ± 5.0 mSv (5.4 to 27.8 mSv) for the scans employing a diagnostic technique.

The mean ± SD (range) effective dose per unit effective mAs from all exams was 0.138 ± 0.046 mSv/mAs (0.036 to 0.445 mSv/mAs) for all patients, from standard CT was 0.134 ± 0.028 mSv/mAs (0.067 to 0.185 mSv/mAs), and from diagnostic CT was 0.142 ± 0.056 mSv/mAs (0.036 to 0.445 mSv/mAs).

The mean ± SD (range) effective dose per unit dose-length product (DLP) from all exams was 0.018 ± 0.046 mSv/mGy-cm (0.036 to 0.445 mSv/mGy-cm) for all patients.

CT radiation doses are summarized in Table 6.

**Table 6:** CT effective dose summary for all patients

CT technique description	DLP	mAs	Effective dose	Effective dose per unit mAs	Effective dose per unit DLP
	(mGy cm)	(mAs)	(mSv)	(mSv/mAs)	(mSv/mGy cm)
Standard	438 ± 84.8	39 ± 11	7.9 ± 1.5	0.134 ± 0.028	0.013 ± 0.002
Diagnostic	1050 ± 342	119 ± 51	15.4 ± 5.0	0.142 ± 0.056	0.015 ± 0.0002

Data are the mean ± standard deviation

The five organs with the highest organ equivalent doses from standard CT in all patients, in order of highest to lowest dose, were thyroid, osteogenic cells, thymus, lungs and brain. CT organ equivalent doses are summarized in Tables 3 and 4.

The five organs with the highest organ equivalent doses from diagnostic CT in male patients, in order of highest to lowest dose, were thyroid, osteogenic cells, thymus, brain and lungs, and in female patients thyroid, osteogenic cells, thymus, brain and heart.

### Radiation doses – total dose

The mean ± SD (range) effective dose from the combined PET and CT portions of all PET/CT exams was 19.6 ± 6.1 mSv (11.0 to 34.3 mSv) for all patients, 19.5 ± 2.3 mSv (11.0 to 31.1 mSv) for male patients and 19.1 ± 3.1 mSv (12.8 to 34.3 mSv) for female patients.

The mean ± SD (range) effective dose from the combined PET and CT portions of the exams using standard CT was 14.0 ± 1.3 mSv (11.0 to 17.6 mSv) for all patients, 13.4 ± 1.1 mSv (11.0 to 16.2 mSv) for male patients and 14.7 ± 1.2 mSv (12.8 to 17.6 mSv) for female patients.

The mean ± SD (range) total effective dose from the combined PET and CT portions of the exams using diagnostic CT technique was 24.4 ± 4.3 mSv (14.6 to 34.3 mSv) for all patients, 25.5 ± 3.4 mSv (17.8 to 31.1 mSv) for male patients and 23.4 ± 4.9 mSv (14.6 to 34.3 mSv) for female patients. Total doses are summarized in Tables 7 and 8.

**Table 7:** Effective dose for male and female patients

CT Technique Description	18F-FDG PET		CT		Total	
	(mSv)		(mSv)		(mSv)	
	Male	Female	Male	Female	Male	Female
Standard CT	8.1 ± 1.2	10.1 ± 1.4	5.3 ± 1.0	4.6 ± 0.8	13.4 ± 1.1	14.7 ± 1.2
Diagnostic CT	8.1 ± 0.8	9.9 ± 1.6	17.4. ± 3.7	13.4 ± 5.5	25.5 ± 3.4	23.4 ± 4.9

Data are the mean ± standard deviation

**Table 8:** Effective dose summary for all patients

CT technique description	18F-FDG PET	CT	Total
Standard CT	9.0 ± 1.6	5.0 ± 1.0	14.0 ± 1.3
Diagnostic CT	9.0 ± 1.6	15.4 ± 5.0	24.4 ± 4.3

Data are the mean ± standard deviation in mSv

The five organs with the highest organ equivalent doses from PET/CT in all patients, in order of highest to lowest dose, were bladder, heart, brain, liver and lungs. PET/CT organ equivalent doses are summarized in Tables 3 and 4.

## Discussion

Risk from radiation exposure to the individual patient is important for evaluation of risk/benefit for justification, and relevant to protocol optimization and personnel protection [40]. The doses themselves utilized in the risk estimation are useful for characterizing the patient population and image acquisition practices of a clinic. A dose estimation method can be based on a combination of reference values and patient- and scanner-specific data. Accurate estimation of patient dose, rather than a conservatively high estimate, is important for many oncology patients who can potentially have many scans and must track cumulative radiation exposure. The accuracy of the patient dose used in the estimation of risk depends on the dose estimation method employed: dose estimated based on reference values depends on the characteristics of the patient population in comparison with the reference population, while the accuracy of the dose estimated based on specific information depends on the extent to which specific information is utilized. Exam- and patient-specific factors are accounted for in an ideal approach to dose estimation. In the current study, we assessed radiation dosimetry of PET/CT for all organs using patient- and exam-specific data and commonly available dosimetry resources.

Effective dose is commonly used when evaluating relative biologic risk, and the Monte Carlo–based organ dose coefficients should not be used to calculate effective dose for individual patients [6, 8]. The International Organization for Medical Physics, ICRP, Health Physics Society and American Association of Physicists in Medicine (AAPM) all have issued policy statements that note the dangers of extrapolating biological risk estimates for radiation doses less than 100 mSv [6, 9–11]. The establishment and use of risk coefficients to estimate public health determinants from individual or population exposures must be considered in the context of uncertainties in the estimates [12]. These uncertainties include dosimetric uncertainties, epidemiological and methodological uncertainties, uncertainties from low statistical power and precision in epidemiology studies of radiation risk, uncertainties in modeling risk data, generalization of risk estimates across different populations and dose rates, as well as reliance on epidemiological studies on observational rather than experimental data [13]. Uncertainties in such risk estimates have been suggested as being up to a factor of 3 lower or higher than the value itself [14]. These rather large uncertainties cause predictions of radiation induced cancers or detriment to be susceptible to biases and confounding influences that are unidentifiable. With due recognition of the limitations imposed by the uncertainties inherent in correlating dose with risk, effective dose is a useful approximation for relating patient population characteristics and evaluating optimization efforts in addition to the clinical benefits of exams.

For internal absorbed dose assessment, OLINDA\EXM was used to perform dosimetry calculations for the various body organs [32]. This code allows calculations for 814 radionuclides and a wide variety of adult, pediatric and pregnant female phantoms; furthermore, it also allows users to modify organ masses in the phantoms for more patient-specific dose calculations. Organ doses calculated by OLINDA\EXM based on reference phantoms representing the average patient were found to be in good agreement with patient specific Monte Carlo mean dose estimates [41]. Monte Carlo results suggest that the difference between the organ equivalent dose estimates in our calculations and those derived from the use of earlier stylized MIRD-type phantoms can be different by a margin as great as 277 %[42]. Patient-specific organ masses were derived from previous reports on variations in the mass of different body organs in relation to stature and BMI ([37–39]. Marine et al, and Clark et al described phantoms that model different body types in a series of percentile height phantoms to evaluate the variation of specific absorbed fractions with height and weight differences across the human population [37, 39]. With patient-specific data and a mean injected activity of 454 MBq, the mean effective dose of 9.0 mSv is consistent with dose per unit injected activity reported in the literature, and also with the dose estimates from ICRP publication 106 [23, 25–28, 34]. The relatively higher injected activity in our clinic at the time of the study results in a relatively higher whole body dose than is observed in other clinics with lower fixed standard injection activity or body mass adjusted injection activity. Despite a lack of 18F-FDG uptake in lung, this organ received the third-highest organ dose according to our methods, and this is most likely due to the proximity of the lung to the heart. Injected activity, patient body mass and height, all available from the patient record, were among the specific factors employed in our estimation of specific internal dose from 18F-FDG.

ImPACT Dose is a readily available CT organ dose calculation tool that reports organ doses based on modeled radiation transport data specific to the CT scanner and represents improved accuracy over previous methods [17]. Without using patient-specific inputs to ImPACT, organ dose was overestimated by 17 %. A contributing factor to this difference is likely the relatively larger size of our patient population. The mean BMI of our patient population was 26.4 while a reference BMI from ICRP Reference Man characteristics is 23.5. It should be noted that there is an opportunity to make the CT organ dose estimate more specific than in our study. Because the Dose Length Product (DLP) in the exam dose report is itself a general value based on a single phantom, the dose estimate may be made more specific by modifying CTDIvol to account for patient size [43]. Scanner-specific results such as the average scan length are useful for identifying opportunities for optimization and for evaluation against similar protocols and standards. For example, the scan length for a whole-body PET/CT can be compared to the scan length for a whole body CT at the same clinic or to a reference value. The average ± SD scan length of diagnostic whole body PET/CT in the present study is 89.5 ± 14.7 cm, while the combined scan length of chest + abdomen + pelvis CT scans is reported as 73 cm [29]. Knowledge of the relative scan lengths puts differences in dose and DLP in perspective. Further, the anatomy in a diagnostic PET/CT is fixed to PET anatomy selection, while the anatomy during the CT-only acquisition may be optimally adjusted. Anatomy selection, and avoidance, is an important aspect of CT protocol optimization that may be overlooked for incorporation into PET/CT scanners. Individual technologists may incorporate optimal anatomy selection in both CT and PET/CT scans, but this optimization measure is more likely to be routinely incorporated as a matter of policy for CT scanners than PET/CT scanners utilized for diagnostic PET/CT exams. In these ways, scanner-specific data are useful for characterizing the clinic’s practices and identifying opportunities for optimization.

Although the average diagnostic PET/CT scan length in this study is greater than that reported in the literature, the dose from the CT portion of the diagnostic PET/CT exams we evaluated in this study is consistent with the average whole body CT dose reported in the literature. Table 9 summarizes doses reported in the literature for whole body CT and chest-abdomen-pelvis (CAP) CT exams, which have approximately the same length scans as whole body PET/CT scans [22, 24, 29–31].

**Table 9:** Comparison of reported whole body diagnostic CT effective dose

CT study	1^a^	2^b^	3^c^	4^d^	5^e^	Average	This study
Effective dose (mSv)	19	9.25	12.5	22.5	16	15.1	15.4

Data are mean mSv

^a^CT-based transmission scan in high quality mode [31]

^b^73-cm CAP CT [29]

^c^Whole body CT, C3 to symphisis pubis [22]

^d^Complete whole body CT includes thyroid contribution [24]

^e^Based on UK survey of CAP CT [30]

The average value of the five included studies reporting whole-body or equivalent CT dose is 15.1 mSv and likely represents a conservative estimate of average dose from a whole body diagnostic CT. A clear advantage of a combined diagnostic PET/CT is the higher patient throughput relative to separate scheduling, along with a relatively shorter exam time than the lower-dose alternatives. From the patient’s perspective, a single visit to the imaging clinic is easier than two separate visits. If the doses are the same, then a combined scan has the additional patient benefit convenience. Depending on the contributing factors at a given imaging clinic, the radiation dose from a standard PET/CT with separate diagnostic CT may be more or less than the radiation dose from a diagnostic PET/CT. Intuitively, a PET/CT with diagnostic technique compared to a standard PET/CT with separately acquired diagnostic CT may appear to be qualitatively equivalent but dosimetrically different because the PET/CT with diagnostic technique CT can also be used for attenuation correction. CT dose from the standard PET/CT appears to be in addition to the dose from the separate diagnostic CT. However, differences in PET/CT and CT scanner hardware and software can cause the dose to be higher or lower. For example, the number of slices is typically different between dual- and single-modality units, and adaptive filtering may not available on PET/CT machines. Both hardware and software factors can cause the patient dose from a combined scan to be higher or lower than separately acquired scans and must be considered when evaluating the dose at a given clinic with reference or literature doses. Less variation is observed in reported PET dose that is estimated from a single source, such as ICRP 106. Even the standards at an imaging clinic for uptake time and acquisition time can affect dose by influencing injected activity. A busy clinic with a fixed injected activity per patient, for example, may administer relatively more activity to account for the inevitable changes in scheduling that come with patient cancellations, delays and other impacts on timing. There is evidence, however, that weight- or BMI-adjusted injected activity reduces patient dose and staff exposure without compromising image quality [44]. A busy clinic may also benefit from a relatively higher injected activity to shorten the acquisition time, as PET acquisition is based on total counts in a bed field and more activity results in the total counts achieved relatively sooner.

The potential for variation in reported dose from PET is apparent in the variability in those PET exam parameters that depend on clinical setting. The patient dose in a clinic employing Germanium-based attenuation correction will differ from a clinic that uses CT-based attenuation correction. Germanium-based attenuation correction has been found to result in negligible patient dose, but it also results in lower diagnostic value in terms of anatomical delineation and longer scanning time than CT-based attenuation correction [31]. The additional challenge of coregistering separate image sets also accompanies this approach. Nonetheless, Germanium-based attenuation correction is sufficient for standard PET/CT exams and still finds routine usage amongst clinics that wish to add PET imaging, but already have a CT scanner. The possibility of different attenuation correction methods makes a one-size-fits-all estimate of expected radiation dose for PET/CT exams unrealistic, and promotes a specific approach to dose evaluation.

Brix et al evaluated total dose from PET/CT exams utilizing CT-based attenuation correction at different hospitals and demonstrated that regardless of whether the PET/CT protocol is standard or diagnostic, the dose from a PET/CT exam is about 23 mSv, due to different approaches, hardware, standards and clinical objectives [23]. RPOP reference doses are compared with the results of our study and with typical literature data in Table 10.

**Table 10:** Comparison of reference dose with our results and literature

	RPOP	This study	Literature^a^
Injected activity (MBq)	400	454	370
PET dose	8	9	7
Standard CT dose	7	5	2.7
Diagnostic CT dose	30	15.4	16.1
Total dose, standard PET/CT	15	14	9.7
Total dose, Diagnostic PET/CT	38	24.4	23.1

Note: doses are in mSv. ^a^Brix et al [23]

The range of doses observed in Table 10 demonstrates the importance of careful choice of data, characterizing the dose at one’s own clinic and understanding the factors contributing to patient dose from PET/CT.

Dose estimated using reference values and standard body models requires the least amount of effort and results in a non-specific estimate that may not be consistent with the characteristics of the actual patient population. However, many exam-specific parameters are conveniently accessed with minimal effort. Reference DLP values are available, but in practice the actual DLP from the exam is readily available from the dose report and is easily used to estimate effective dose by employing either a standard or scanner-specific factor [18]. An approach employing all available patient-specific resources (i.e. patient-adjusted DLP and organ mass, scanner-specific factors) results in accurate estimation but requires moderate effort because this data must be retrieved from electronic records. A retrospective dose estimate requires accessing DICOM information and patient records for relevant information, while estimating dose from a present exam for an individual patient can utilize information at hand. Whether the risk estimate is made at the time of the exam or retrospectively, and whether it is for an individual or representative group, the effort involved in gathering the information must be weighed against the value of the information gained.

## Conclusions

An ideal dose estimation method is convenient to employ and considers clinic- and patient-specific factors to an appropriate extent. Patient specific parameters (e.g., weight, and height) identified from patient data allow for more accurate estimate of organ equivalent doses and the effort involved in obtaining the data is worthwhile for a retrospective analysis of a representative patient sample and for some individual patients. Accurate estimation of patient dose, rather than a conservatively high estimate, is important for many oncology patients who can potentially have many scans and must track cumulative exposure. Considering actual patient-specific characteristics resulted in lower organ equivalent dose estimates than traditional reference phantoms and models, but the methods employed in this study resulted in organ doses and effective doses that are in agreement with alternative methods. Considering the substantial radiation dose of whole body PET/CT exams and the potential for wide variation in contributing factors, all aspects of the exam process should be considered for optimization. Radiopharmaceutical injection activity, CT technique, CT protocol optimization measures, anatomy selection, and convenience to both patient and imaging clinic are all factors which must be considered for comprehensive optimization and subsequent justification of the PET/CT exam. The effort involved in obtaining patient-specific data is reasonable to help justify the risk associated with the radiation exposure, optimize the image acquisition parameters to balance dose with the objectives of the exam, and characterize the patient population.

Clinic-specific patient dose for each type of PET/CT exam can reasonably be estimated for comparison with industry standards, reference values and literature values. Opportunities for optimization may also be apparent in better understanding how the clinic’s doses compare with other sources of data.

## Abbreviations

AAPM, American Association of Physicists in Medicine; BMI, Body mass index; ICRP, International Commission on Radiological Protection; PET/CT, Fluorine-18 positron emission tomography/computed tomography; RPOP, Radiation protection of patients
